# Supplementary material for: Ultraviolet radiation drives mutations in a subset of mucosal melanomas
Source: Nat Commun. 2021 Jan 11;12:259. doi: 10.1038/s41467-020-20432-5 (PMC7801393; doi:10.1038/s41467-020-20432-5)
Supplement: Supplementary file 1 — Supplementary Information [file 41467_2020_20432_MOESM1_ESM.pdf]

Ultraviolet radiation drives mutations in a subset of mucosal melanomas

Piyushkumar A. Mundra[1†], Nathalie Dhomen[1†], Manuel Rodrigues[2,3], Lauge Hjorth Mikkelsen[4], Nathalie Cassoux[5], Kelly Brooks[1,6], Sara Valpione[1,7], Jorge S. Reis-Filho[8], Steffen Heegaard[4], Marc-Henri Stern[2,9], Sergio Roman-Roman[10], and Richard Marais[1,*]

[1]*Molecular Oncology Group, Cancer Research UK Manchester Institute, The University of Manchester, Alderley Park, SK10 4TG, UK*
[2]*Institut Curie, PSL Research University, INSERM U830, DNA Repair and Uveal Melanoma (D.R.U.M.), Equipe labellisée par la Ligue Nationale contre le Cancer, Paris 75248, France.*
[3]*Institut Curie, PSL Research University, Department of Medical Oncology, Paris 75248, France.*
[4]*Department of Pathology/Eye Pathology Section, University of Copenhagen, Rigshospitalet, 2100 Copenhagen, Denmark*
[5]*Institut Curie, PSL Research University, Department of Ocular Oncology, Paris 75248, France.*
[6]*QIMR Berghofer Medical Research Institute, Brisbane, Queensland, 4006, Australia*
[7]*The Christie NHS Foundation Trust, Manchester, M20 4GJ, UK*
[8]*Experimental Pathology Service, Department of Pathology, Memorial Sloan Kettering Cancer Center, USA*
[9]*Institut Curie, PSL Research University, Department of Genetics, Paris 75248, France*
[10]*Institut Curie, PSL Research University, Translational Research Department, Paris 75248, France*

Running Title: UVR exposure is a dominant mutational process in mucosal melanoma

Keywords: UVR, mucosal melanoma, conjunctival melanoma, mutational signature

[†]These authors contributed equally to this work.

[*]**Correspondence:**
Richard Marais, PhD
Cancer Research UK Manchester Institute
The University of Manchester
Alderley Park
SK10 4TG, UK
Email: richard.marais@cruk.manchester.ac.uk

SUPPLEMENTARY INFORMATION

Patient ID	Age range	Tumour location	Tumour Stage	Histology	Months to death or last follow-up	Cohort
MuM1	<50	Fornix	T2b	Mixed	82	Rigshospitalet
MuM10	>60	Extended PAM lesions including inferior and superior tarsal conjunctivae until fornices. Sample came from the superior fornix	T3b	Epithelioid	73	Curie
MuM11	>60	Tarsal conjunctiva extending onto the lid margin	T2b	Mixed	30	Rigshospitalet
MuM12	50-60	Limbus	T1b	Epithelioid	48	Curie
MuM13	<40	Limbus	T1a	Epithelioid	46	Rigshospitalet
MuM14	>70	Upper part of the bulbar conjunctiva	T1a	Mixed	58	Rigshospitalet
MuM15	>80	Large lesion from superior fornix (not exposed) to caruncle (exposed)	T3b	Epithelioid	59	Curie
MuM16	<40	Limbus	T2b	Epithelioid	52.6	Curie
MuM17	>60	Limbus	T1c	Epithelioid	16.9	Curie
MuM18	>80	Tarsal conjunctiva superior	T1a	NS	3	Curie

Supplementary Table 1

Clinical characteristics for 10 conjunctival melanoma patients.

Patient ID	Donor ID	Sample ID	Specimen Type	Primary Site
MuM5	MELA_0204	EXTERN_MELA_20140528_006	Primary	Vulva
MuM3	MELA_0013	EXTERN_MELA_20140505_023	Primary	Vulva
MuM9	MELA_0056	EXTERN_MELA_20140514_026	Regional Lymph Nodes	Nasal Cavity
MuM4	MELA_0058	EXTERN_MELA_20140514_030	Primary	Vulva
MuM8	MELA_0059	EXTERN_MELA_20140514_032	Primary	Nasal Cavity
MuM2	MELA_0063	EXTERN_MELA_20140514_041	Primary	Other - Lt lacrimal sac
MuM7	MELA_0065	EXTERN_MELA_20140514_045	Primary	Rectum
MuM6	MELA_0071	EXTERN_MELA_20140514_058	Primary	Vagina

Supplementary Table 2

Clinical characteristics for the 8 published[1] mucosal melanoma patients.

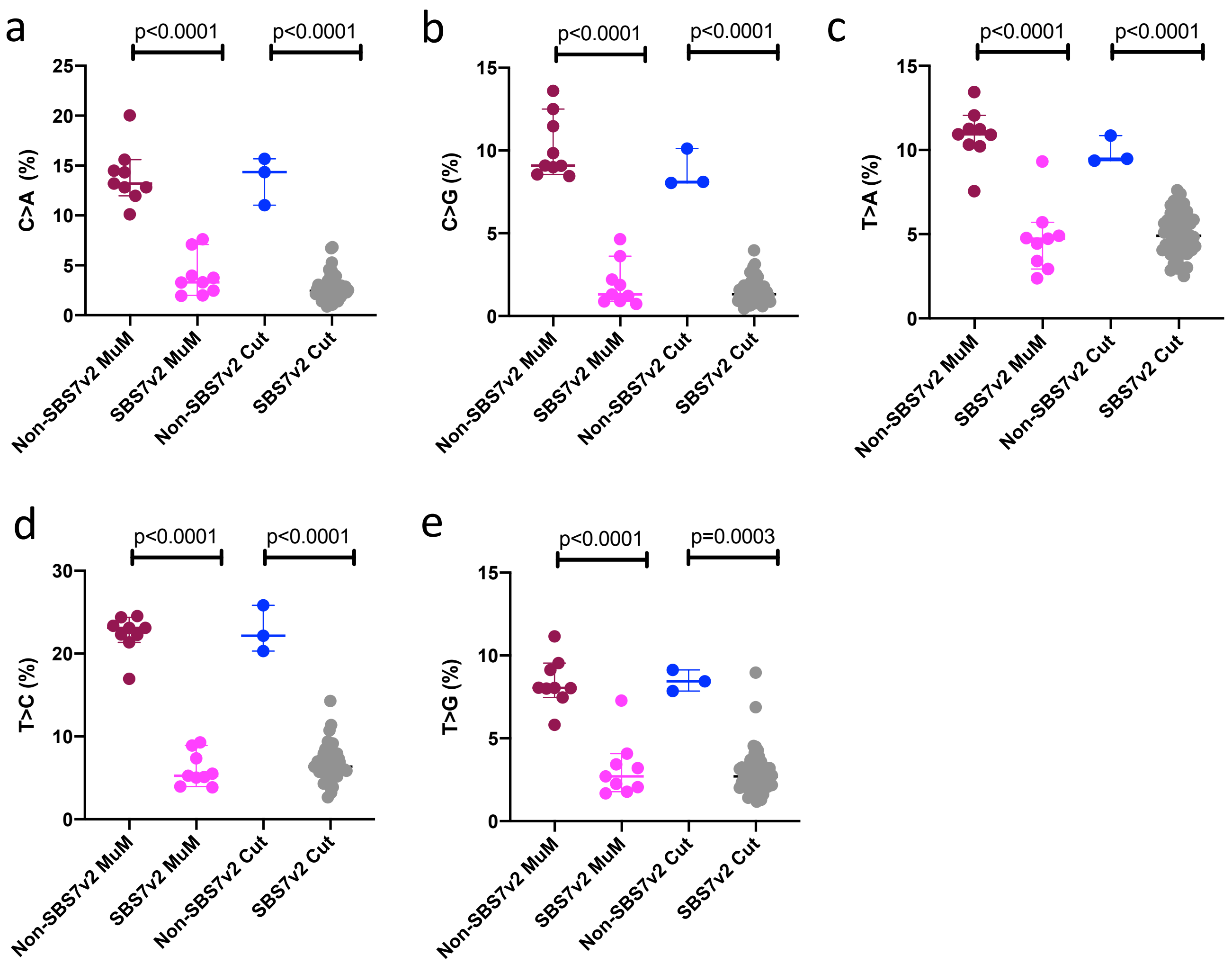

Supplementary Figure 1: Nucleotide transitions in mucosal and common cutaneous melanomas.

a-e Proportions of each of the five nucleotide transitions/transversions C>A (**a**), C>G (**b**), T>A (**c**), T>C (**d**), and T>G (**e**) in the genomes of mucosal (MuM) and cutaneous (Cut) melanomas with SBS7v2-dominant (magenta n= 9, grey n=51) and non-SBSv2 (maroon n=9, blue n=3) genomes. All panels show median and 95% confidence intervals, dots denote individual tumors, p-values determined by two-tailed Mann Whitney U.

Supplementary Figure 2: Analysis of publicly available WGS[2].

a Mutation signatures weighted by relative contribution to spectrum of mutations occurring in 65 published mucosal melanoma genomes[2]. Index above indicates subdivision into non-SBS7v2 (blue, n=57) and SBS7v2-dominant (magenta, n=8) genomes. Columns represent individual tumors. **b** Total SNVs in published[2] non-SBS7v2 (maroon, n=57) and SBS7v2-dominant (magenta, n=8) mucosal melanomas (p<0.0001). **c-h** Proportions of each of the six nucleotide transitions/transversions C>T (**c**, p<0.0001), C>A (**d, p**<0.0001), C>G (**e**, p<0.0001), T>C (**f**, p<0.0001), T>A (**g**, p<0.0001), and T>G (**h**, p<0.0001) in the published[1] genomes of non-SBS7v2 (maroon, n=57) and SBS7v2-dominant (magenta, n=8) mucosal melanomas. Panels (**b-h**), show median and 95% confidence intervals, dots denote individual tumors, p-values determined by two-tailed Mann Whitney U.

Supplementary Figure 2

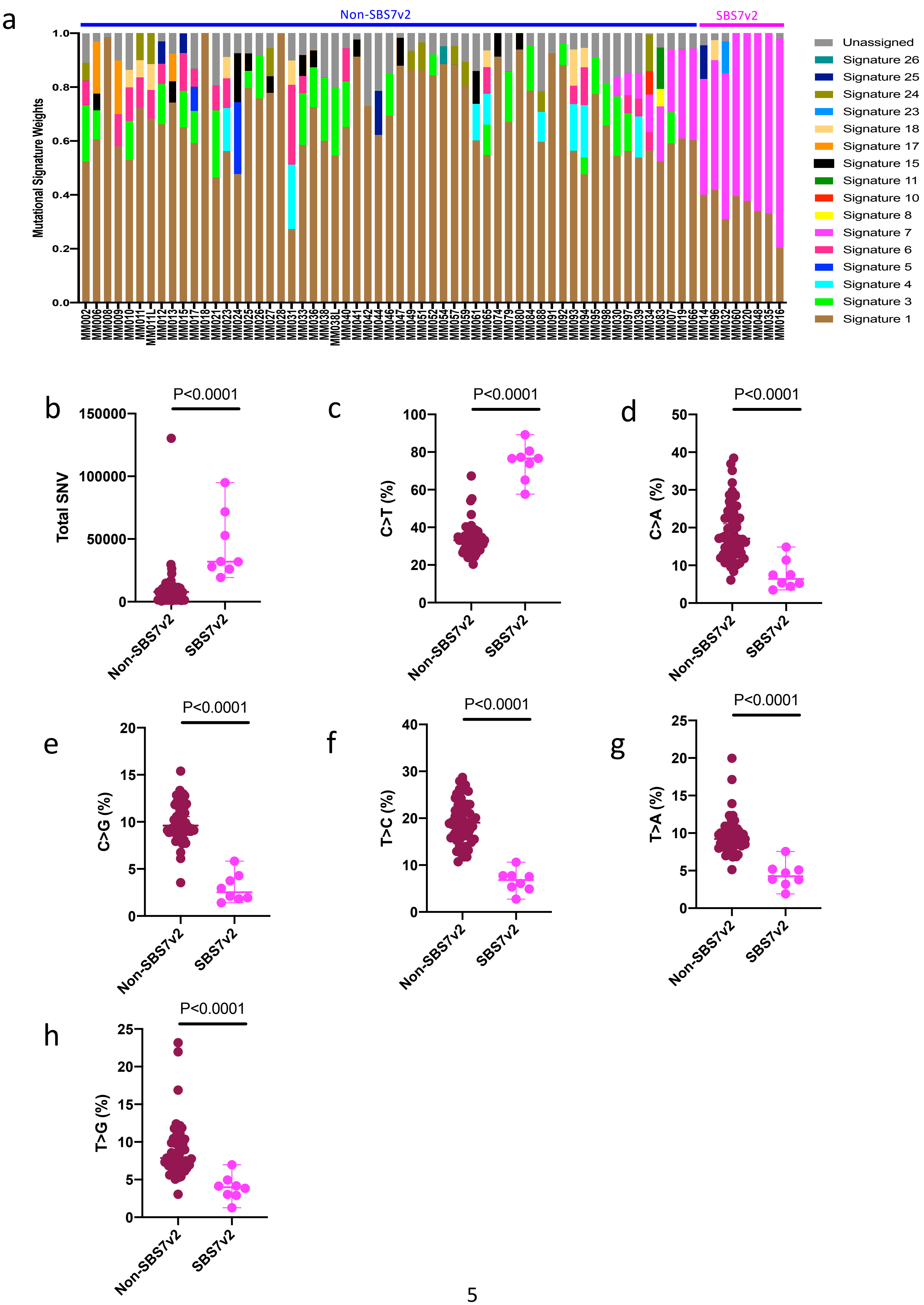

Supplementary Figure 3

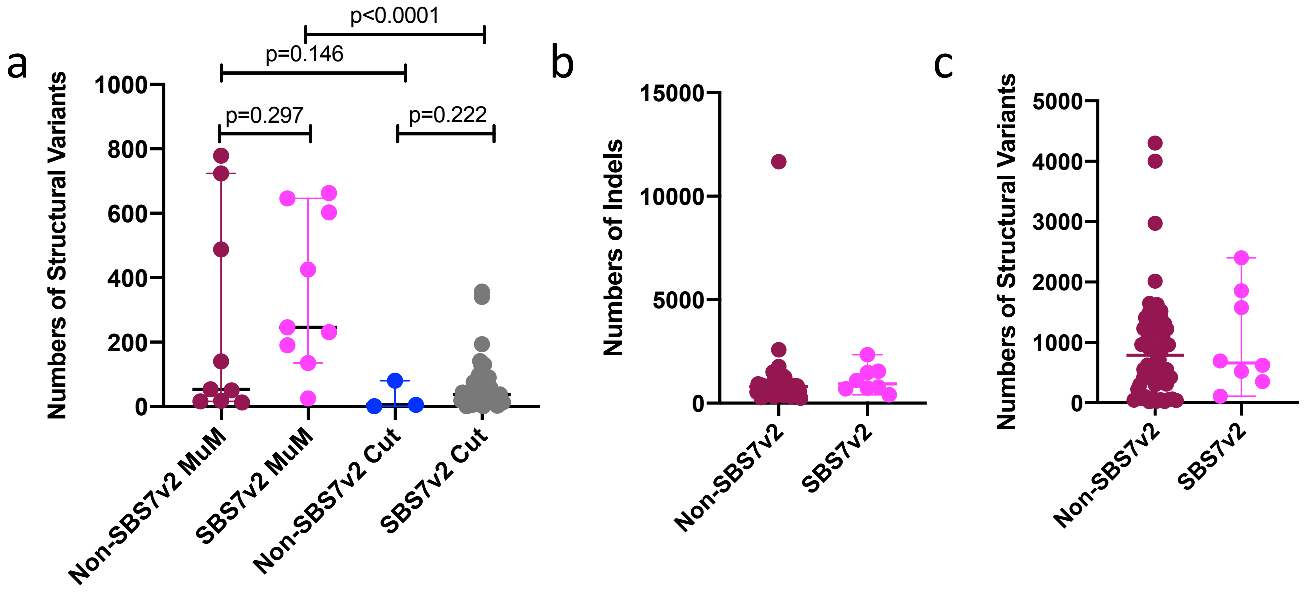

Supplementary Figure 3: Structural variants in mucosal and common cutaneous melanomas
a Numbers of structural variants in mucosal (MuM) and cutaneous (Cut) melanomas with SBS7v2-dominant (magenta n=9, grey n=51, respectively) or non-SBSv2 (maroon n=9, blue n=3) genomes. p=0.297 between Non-SBS7v2 MuM and SBS7v2 MuM, p=0.222 between Non-SBS7v2 Cut and SBS7v2 Cut, p=0.146 between Non-SBS7v2 MuM and Non-SBS7v2 Cut, p<0.0001 between SBS7v2 MuM and SBS7v2 Cut. **b,c** Numbers of indels and (**b**) and structural variants (**c**) in published[2] non-SBS7v2 (maroon, n=57) and SBS7v2-dominant (magenta, n=8) mucosal melanomas. All panels show median and 95% confidence intervals, dots denote individual tumors, p-values determined by two-tailed Mann Whitney U.

References

1 Hayward, N. K. *et al.* Whole-genome landscapes of major melanoma subtypes. *Nature* **545**, 175-180, doi:10.1038/nature22071 (2017).

2 Zhou, R. *et al.* Analysis of Mucosal Melanoma Whole-Genome Landscapes Reveals Clinically Relevant Genomic Aberrations. *Clin Cancer Res* **25**, 3548-3560, doi:10.1158/1078- 0432.CCR-18-3442 (2019).
