# Supplementary material for: Ultraviolet radiation drives mutations in a subset of mucosal melanomas
Source: Nat Commun. 2021 Jan 11;12:259. doi: 10.1038/s41467-020-20432-5 (PMC7801393; doi:10.1038/s41467-020-20432-5)
Supplement: Supplementary file 3 — Description of Additional Supplementary Files [file 41467_2020_20432_MOESM3_ESM.pdf]

Description of Additional Supplementary Files

File Name: Supplementary Data 1

Description: Supplementary Data 1: Summary of WGS data for mucosal and common cutaneous melanoma patient samples (directly sequenced or previously published) reported in this study.
