# Supplementary material for: Ultraviolet radiation drives mutations in a subset of mucosal melanomas
Source: Nat Commun. 2021 Jan 11;12:259. doi: 10.1038/s41467-020-20432-5 (PMC7801393; doi:10.1038/s41467-020-20432-5)
Supplement: Supplementary file 5 — Reporting Summary [file 41467_2020_20432_MOESM5_ESM.pdf]

nature research

Corresponding author(s): Prof Richard Marais

Last updated by author(s): Nov 10, 2020

Reporting Summary

Nature Research wishes to improve the reproducibility of the work that we publish. This form provides structure for consistency and transparency in reporting. For further information on Nature Research policies, see our Editorial Policies and the Editorial Policy Checklist.

Statistics

For all statistical analyses, confirm that the following items are present in the figure legend, table legend, main text, or Methods section.

n/a	Confirmed	
☐	☒	The exact sample size (*n*) for each experimental group/condition, given as a discrete number and unit of measurement
☒	☐	A statement on whether measurements were taken from distinct samples or whether the same sample was measured repeatedly
☐	☒	The statistical test(s) used AND whether they are one- or two-sided<br>*Only common tests should be described solely by name; describe more complex techniques in the Methods section.*
☒	☐	A description of all covariates tested
☒	☐	A description of any assumptions or corrections, such as tests of normality and adjustment for multiple comparisons
☐	☒	A full description of the statistical parameters including central tendency (e.g. means) or other basic estimates (e.g. regression coefficient) AND variation (e.g. standard deviation) or associated estimates of uncertainty (e.g. confidence intervals)
☐	☒	For null hypothesis testing, the test statistic (e.g. *F*, *t*, *r*) with confidence intervals, effect sizes, degrees of freedom and *P* value noted<br>*Give P values as exact values whenever suitable.*
☒	☐	For Bayesian analysis, information on the choice of priors and Markov chain Monte Carlo settings
☒	☐	For hierarchical and complex designs, identification of the appropriate level for tests and full reporting of outcomes
☒	☐	Estimates of effect sizes (e.g. Cohen's *d*, Pearson's *r*), indicating how they were calculated

Our web collection on statistics for biologists contains articles on many of the points above.

Software and code

Policy information about availability of computer code

Data collection	Whole genome sequencing BAM files of 8 primary mucosal melanomas and primary cutaneous melanomas were downloaded using ASPERA (v3.5.4) from EGA using accession ID EGAS00001001552. FASTQ files were subsequently extracted for these BAM files using samtools2 (v1.3.1).
Data analysis	The 2 x 150 reads were mapped to the reference genome GRCh37 (v75) using BWA-mem (v0.7.7). Following duplicate removal using PICARD (v1.96) and INDELs realignment and recalibration of base qualities using GATK (v3.6), the somatic variant calling was performed using MUTECT (v1.1.7) with default parameters. Small insertions and deletions were determined using Strelka (v1.0.4). Variant effect predictor (Ensembl version 73) was used to annotate the mutations. Structural variants were determined using DELLY (v0.8.1) with default parameters. Mutational signatures were determined using deconstructSigs (v1.8.0) package using default parameters. Copy number alterations were determined using Sequenza (v2.1.9999b0) package with parameters (mufreq.treshold = 0.05, min.reads = 10, min.fw.freq = -0.1).<br><br>In-house script used to calculate fraction of genome altered is available at: https://github.com/mpiyush21/MucosalNatureComms

For manuscripts utilizing custom algorithms or software that are central to the research but not yet described in published literature, software must be made available to editors and reviewers. We strongly encourage code deposition in a community repository (e.g. GitHub). See the Nature Research guidelines for submitting code & software for further information.

Data

Policy information about availability of data

All manuscripts must include a data availability statement. This statement should provide the following information, where applicable:

- Accession codes, unique identifiers, or web links for publicly available datasets
- A list of figures that have associated raw data
- A description of any restrictions on data availability

Whole-genome sequencing data from conjunctival melanoma samples has been deposited with the EGA under accession number EGAS00001004697 (https://www.ebi.ac.uk/ega/studies/EGAS00001004697). Cutaneous melanoma and other mucosal melanoma were submitted by Hayward et al, 2017 and are available with EGA accession ID EGAS00001001552 (https://www.ebi.ac.uk/ega/studies/EGAS00001001552).

Field-specific reporting

Please select the one below that is the best fit for your research. If you are not sure, read the appropriate sections before making your selection.

- [x] Life sciences
- [ ] Behavioural & social sciences
- [ ] Ecological, evolutionary & environmental sciences

For a reference copy of the document with all sections, see nature.com/documents/nr-reporting-summary-flat.pdf

Life sciences study design

All studies must disclose on these points even when the disclosure is negative.

Sample size	We perform whole genome sequencing analysis on 10 primary mucosal (conjunctival) tumor samples and sourced other 8 primary mucosal melanoma and 54 primary cutaneous melanoma samples from the published study. Given that conjunctival melanomas are extremely rare subtype and the sample size is limited by availability of the appropriate samples, we recruited as many samples as possible for our study.
Data exclusions	None of the data points were excluded.
Replication	This is a genomic sequencing study. Given the prevalent standard practice, each sample was only sequenced once. Each tumor sequence was assumed as an independent biological replicate. Further, the conjunctival melanoma cohort presented in this study is the largest cohort of such a rare type of cancer and hence replication in another cohort is not possible at this time.
Randomization	Randomization is not appropriate given the study objective and the nature of the whole genome sequencing analysis of a rare cancer type.
Blinding	Clinical information was blinded from the bioinformatician performing computational analysis.

Reporting for specific materials, systems and methods

We require information from authors about some types of materials, experimental systems and methods used in many studies. Here, indicate whether each material, system or method listed is relevant to your study. If you are not sure if a list item applies to your research, read the appropriate section before selecting a response.

Materials & experimental systems

n/a	Involved in the study	
x		Antibodies
x		Eukaryotic cell lines
x		Palaeontology and archaeology
x		Animals and other organisms
	x	Human research participants
x		Clinical data
x		Dual use research of concern

Methods

n/a	Involved in the study	
x		ChIP-seq
x		Flow cytometry
x		MRI-based neuroimaging

Human research participants

Policy information about studies involving human research participants

Population characteristics	Median patient age 66 years, range 38-84; 6 females, 4 males; Supplementary Table 1
Recruitment	Due to the rarity of the condition described, patients were recruited on an ad hoc basis as they presented to the clinic. Both clinics are located in Western Europe and thus in comparing the 10 conjunctival samples with published mucosal melanomas from an Australian study we are also comparing two geographically distinct cohorts. However, given the Australian cohort would be considered to have a higher exposure to UVR overall, and that the samples from the Western European cohort exhibited greater evidence of UVR-related damage at the genomic level, suggests that it is UVR exposure at a given body site rather than location of the cohort population that determines the outcomes observed in this study.
Ethics oversight	Conjunctival melanoma samples MuM1 and MuM10-18 comprise two cohorts, one from Institut Curie, Paris (MuM10, MuM12, MuM15-18) and one from the department of Pathology/Eye Pathology Section, University of Copenhagen, Rigshospitalet (MuM1, MuM11, MuM13, MuM14). The studies were approved by the Internal Review Board of Institut Curie (2014) and the Danish National Ethics Committee (j.no. 1700673) respectively.

Note that full information on the approval of the study protocol must also be provided in the manuscript.
